# Regulation of auditory plasticity during critical periods and following hearing loss

**DOI:** 10.1016/j.heares.2020.107976

**Published:** 2020-11

**Authors:** Dora Persic, Maryse E. Thomas, Vassilis Pelekanos, David K. Ryugo, Anne E. Takesian, Katrin Krumbholz, Sonja J. Pyott

**Affiliations:** a University of Groningen, University Medical Center Groningen, Groningen, Department of Otorhinolaryngology and Head/Neck Surgery, 9713, GZ, Groningen, the Netherlands; b Eaton-Peabody Laboratories, Massachusetts Eye & Ear and Department of Otorhinolaryngology and Head/Neck Surgery, Harvard Medical School, Boston, MA, USA; c Hearing Sciences, Division of Clinical Neuroscience, School of Medicine, University of Nottingham, University Park, Nottingham, UK; d Hearing Research, Garvan Institute of Medical Research, Sydney, NSW, 2010, Australia; e School of Medical Sciences, UNSW Sydney, Sydney, NSW, 2052, Australia; f Department of Otolaryngology, Head, Neck & Skull Base Surgery, St Vincent’s Hospital, Sydney, NSW, 2010, Australia

**Keywords:** Developmental critical periods, Sensory deprivation, Auditory cortex, Auditory brainstem, Neuronal reorganization, Hearing loss, Hidden hearing loss, Synaptopathy, Aging, Tinnitus

## Abstract

Sensory input has profound effects on neuronal organization and sensory maps in the brain. The mechanisms regulating plasticity of the auditory pathway have been revealed by examining the consequences of altered auditory input during both developmental critical periods—when plasticity facilitates the optimization of neural circuits in concert with the external environment—and in adulthood—when hearing loss is linked to the generation of tinnitus. In this review, we summarize research identifying the molecular, cellular, and circuit-level mechanisms regulating neuronal organization and tonotopic map plasticity during developmental critical periods and in adulthood. These mechanisms are shared in both the juvenile and adult brain and along the length of the auditory pathway, where they serve to regulate disinhibitory networks, synaptic structure and function, as well as structural barriers to plasticity. Regulation of plasticity also involves both neuromodulatory circuits, which link plasticity with learning and attention, as well as ascending and descending auditory circuits, which link the auditory cortex and lower structures. Further work identifying the interplay of molecular and cellular mechanisms associating hearing loss-induced plasticity with tinnitus will continue to advance our understanding of this disorder and lead to new approaches to its treatment.

## Highlights

• During CPs, brain plasticity is enhanced and sensitive to acoustic experience.
• Enhanced plasticity can be reinstated in the adult brain following hearing loss.
• Molecular, cellular, and circuit-level mechanisms regulate CP and adult plasticity.
• Plasticity resulting from hearing loss may contribute to the emergence of tinnitus.
• Modifying plasticity in the adult brain may offer new treatments for tinnitus.

## 1 Overview

A remarkable feature of sensory pathways in the brain is their organization of sensory representations in the form of topographic maps. In the auditory domain, frequency receptive fields of neurons form an orderly tonotopic map. Tonotopy is preserved along the auditory neuraxis and reflects the topographically organized projections from the cochlea. Organization of the tonotopic map is genetically predetermined and laid out coarsely during prenatal development. It is subsequently refined in an activity-dependent manner, first by spontaneous activity and, following hearing onset, by evoked activity (Clause et al., 2014; Kandler et al., 2009; Tritsch et al., 2010; Sanes and Woolley, 2011; Wang and Bergles, 2015). The crucial role of auditory experience for the proper development of the tonotopic map is evident during critical periods, when the quality of the acoustic environment leaves a robust imprint on the receptive fields of cortical neurons (Zhang et al., 2001). Although organization of the tonotopic map was traditionally considered resistant to alterations following the closure of the critical period, converging evidence indicates retained (albeit reduced) plasticity in the adult brain. This plasticity can be observed at different levels of neuronal organization, ranging from molecular, cellular and synaptic changes in excitability, to shifts in tuning of single neurons, to large-scale, stable reorganization of the tonotopic map. Many aspects of auditory plasticity and their perceptual consequences have been reviewed elsewhere (Irvine, 2018; Keuroghlian and Knudsen, 2007; Pienkowski et al., 2011; Schreiner and Polley, 2014; Syka, 2002; Weinberger, 2007). Here, we focus on the molecular and cellular mechanisms regulating neuronal organization and map plasticity during developmental critical periods and in the adult brain. We emphasize changes in regulation driven by peripheral auditory deprivation, for instance, through hearing loss. We conclude with a discussion of how plasticity and neuronal reorganization—triggered by overt and also hidden hearing loss—may contribute to tinnitus.

## 2 Developmental critical periods in the auditory cortex

Critical periods (CPs) are defined epochs of early postnatal development, when structural and functional neural circuits are shaped by passive experience with the external environment (Hensch, 2004; Kuhl, 2010). These windows of plasticity drive the maturation of subcortical and cortical sensory representations, with consequences for emerging behavior and cognition (Hess, 1958; Lenneberg, 1967; Lorenz, 1935; Scovel, 1969). In the auditory system, this rapid optimization facilitates feature-specific expertise such as phoneme identification, language acquisition, absolute pitch, and musical aptitude (Penhune, 2011; Werker and Hensch, 2015; Zhao and Kuhl, 2016), yet also represents a period of susceptibility to impoverished environments (Bures et al., 2017; Knudsen, 2004; Sanes and Woolley, 2011; Uylings, 2006). For example, prelingual deafness has permanent impact on the development of auditory circuits. Children born deaf may regain hearing with cochlear implants but demonstrate sustained perceptual and language deficits that are more pronounced with delayed implantation (Kral and Sharma, 2012; Kral et al., 2019; Nicholas and Geers, 2006; Svirsky et al., 2004).

**Fig. 1:**
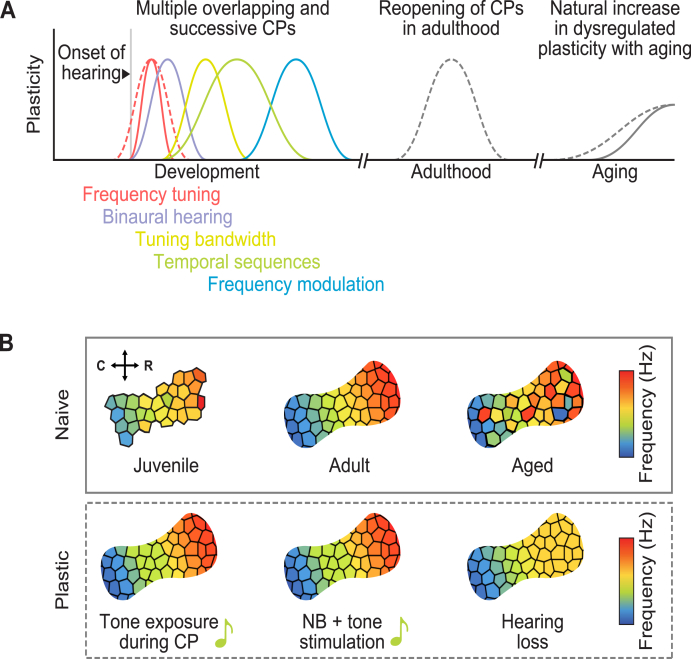
**Auditoryplasticity in development and adulthood inrodentmodels. A.** Auditory development is characterized by multiple overlapping and successive critical periods (CPs) during which sensory representations are formed. CP timing is malleable and depends on the maturation of plasticity regulators. Following maturation, sensory representations are stable, and CPs can only be reopened if molecular brakes are lifted. A natural increase in dysregulated plasticity is observed with aging, which may be accelerated by hearing loss. **B.** Trajectory of tonotopic map development in the primary auditory cortex. Left: The juvenile tonotopic map is characterized by incomplete maturation prior to CP closure. Middle: Mature tonotopic representations form an orderly frequency gradient along the caudal to rostral axis. Right: Tonotopic disorganization has been observed with aging. **C.** Tonotopic map reorganization due to enhanced, CP-like, plasticity. Left: Passive tone pip exposure during the CP for tonotopic map plasticity results in an over-representation of the exposed tone frequency within the tonotopic map. Middle: Frequency over-representation can be induced in the adult cortex by pairing nucleus basalis stimulation with tone presentation. Right: Peripheral hearing loss in a restricted frequency range can result in an over-representation of the spared frequencies.

Plasticity of the cortical tonotopic map is the chief model of CP plasticity in the auditory system. The tonotopic map is established during a CP for frequency tuning, when passive acoustic experiences influence map organization across species (Keuroghlian and Knudsen, 2007; Kral, 2013; Sanes and Woolley, 2011; Werker and Hensch, 2015). In rodents, exposure to pure tones results in the expanded representation of the tone frequency within the tonotopic map (Han et al., 2007; Kim and Bao, 2009; Zhang et al., 2001). This robust plasticity is restricted to a short three-day period, usually reported as postnatal days 11–14, following the maturation of peripheral auditory structures (Barkat et al., 2011; de Villers-Sidani et al., 2007). This plasticity occurs in primary auditory cortex but not auditory thalamus, implicating plasticity at thalamocortical or intracortical synapses (Barkat et al., 2011). CPs have also been described for other sound features including binaural hearing, tuning bandwidth, temporal sequences, frequency modulation, and amplitude modulation (Caras and Sanes, 2015; Insanally et al., 2009; Nakahara et al., 2004; Polley et al., 2013). In general, CPs are thought to occur in a hierarchical sequence, with representations for basic features consolidating before more complex ones (de Villers-Sidani and Merzenich, 2011; Insanally et al., 2009; Sanes and Woolley, 2011). Fig. 1A and B depict this developmental trajectory in the rodent primary auditory cortex.

Interestingly, the timing of CPs is itself plastic and can be influenced by the sensory environment (Takesian and Hensch, 2013; Voss et al., 2017). Unstructured, noisy environments postpone CP closure (Chang and Merzenich, 2003), whereas exposure to highly structured pulsed tones or pulsed white noise hastens CP closure (Zhang et al., 2002; Zhou and Merzenich, 2012). Enriched sensory environments also keep the CP open longer and have opposite effects to deprivation, stimulating dendritic growth and improving auditory response properties (Bose et al., 2010; Bures et al., 2018; Cai et al., 2010). Effects of sound exposure on CP timing can be further restricted to specific functional regions within the auditory cortex. For example, band-limited noise delays tonotopic refinement only for portions of the tonotopic map within the noise band (de Villers-Sidani et al., 2008). The presence of high-fidelity sensory inputs is, therefore, necessary for the normal progression of critical periods and the functional and structural maturation of the primary auditory cortex (de Villers-Sidani and Merzenich, 2011), as has been analogously demonstrated in primary visual cortex (Crair et al., 1998).

The absence of early sensory experience is expected to have the most profound effects on auditory cortical representations. Rodent studies indicate that complete sensorineural hearing loss during development disrupts synaptic inhibition and increases excitability throughout the central auditory pathway, resulting in broadened frequency tuning (reviewed in Sanes and Bao, 2009; Takesian et al., 2009). Neonatal high frequency hearing loss induced through cochlear lesions or traumatic noise exposure alters cortical tonotopy, shifting the tuning preference of neurons in high frequency regions to lower frequencies (Eggermont and Komiya, 2000; Harrison et al., 1991). Even transient hearing loss during development, such as that experienced by children with otitis media, can result in persistent changes in inhibitory synapses (Mowery et al., 2015, 2019; Takesian et al., 2012) that could contribute to sustained perceptual deficits later in life (Caras and Sanes, 2015; Takesian et al., 2012; Whitton and Polley, 2011). However, heightened plasticity during early life may also serve to functionally enhance inputs from spared sensory structures during auditory deprivation (Bavelier and Neville, 2002; Kral, 2007; Sharma et al., 2015), often consistent with improved perceptual abilities in the intact modalities (Lomber et al., 2010; Meredith et al., 2011). This cross-modal plasticity occurs more prominently in higher-order auditory areas as opposed to primary auditory cortex (Kral, 2007) and has been found to preserve the functional role of cortical regions, such as stimulus localization, despite enhancing responses to a novel modality (Meredith et al., 2011).

## 3 Evidence of cortical plasticity in the adult auditory cortex

Just as early sensory experience is necessary for the normal development of sensory brain regions, the continued presence of patterned sensory inputs is also critical for adult brain function. Decades of research have shown that both loss of sensory input from the periphery (e.g., Chino et al., 1992; Diamond et al., 1993; Maier et al., 2003; Robertson and Irvine, 1989) and significant changes in the sensory environment (e.g., Pienkowski and Eggermont, 2009; Zhou et al., 2011) can result in the reorganization of adult sensory cortices across species. For example, seminal studies examining plasticity of the somatosensory cortex in adult nonhuman primates reported that, after finger deafferentation, the deprived cortical territory was invaded by an expansion of the neighboring finger representations (Merzenich et al., 1983a, 1983b; Pons et al., 1991). These findings indicate that the mechanisms that constrain plasticity after the CP can be at least partially released to permit reorganization of sensory representations in the mature cortex. In this section, we highlight evidence that shows the potential for CP-like plasticity in the mature auditory cortex following alteration of acoustic inputs.

Large scale topographic map changes in auditory cortex have been demonstrated following peripheral deafferentation. Restricted damage to the auditory periphery induces robust plasticity in auditory cortex of both juvenile and adult animals (Pienkowski and Eggermont, 2011), mirroring earlier findings in somatosensory cortex. For example, spatially-restricted lesions of the adult cochlea cause an over-representation in primary auditory cortex of the frequencies that stimulate cochlear regions flanking the damage, distorting the cortical tonotopic map (Eggermont and Komiya, 2000; Rajan et al., 1993; Robertson and Irvine, 1989). Acute exposure to intense pure tones also causes a profound reorganization of tonotopic maps in the adult primary auditory cortex, accompanied by the broadening of tuning curves and increases in both spontaneous and sound-evoked activity (Calford et al., 1993; Kimura and Eggermont, 1999; Norena et al., 2003, 2008).

Mounting evidence has shown that even non-traumatic sound experience may result in central auditory plasticity. Extended (week-long) exposure to moderate-intensity tones is associated with reduced neural excitability in the cat auditory cortex (Pienkowski and Eggermont, 2012). This reduction in activity is restricted to the portion of the auditory cortex corresponding to the exposure frequency range and likely reflects homeostatic plasticity in response to chronic stimulation. Prolonged exposure to around-the-clock broadband white noise at non-traumatic intensities has also been found to result in disorganization of tonotopic gradients and disruption of frequency processing in the adult rat primary auditory cortex (Thomas et al., 2019; Zheng, 2012; Zhou and Merzenich, 2012). This phenomenon has been proposed to occur through a reopening of CP plasticity since exposure to pure tones following noise exposure results in their exaggerated representation within the tonotopic map (Thomas et al., 2019; Zhou and Merzenich, 2012). In contrast to CP plasticity, however, sound exposure must be persistent and long-lasting to induce map plasticity in adulthood.

Later in life may also be a period during which sensory representations are unusually plastic. Noisy or absent sensory inputs caused by natural age-related degeneration of the auditory periphery may contribute to dysregulated plasticity in the aged auditory cortex (Kamal et al., 2013). Aging is accompanied by reduced representational stability, poorer temporal processing, and disrupted tonotopy in auditory cortex (Kamal et al., 2013; Mendelson and Ricketts, 2001; Turner et al., 2005). A recent report found that the tonotopic map of aged rats exhibits plastic reorganization in response to passive tone exposure, similar to CP plasticity (Cisneros-Franco et al., 2018). However, unlike the CP, this plasticity does not result in the formation of stable representations, as map reorganization could be easily ‘overwritten’ by a second pure tone exposure. While aging has long been associated with a loss of neural plasticity, this study instead suggests that there is enhanced, but dysregulated, plasticity in the aged brain, which may have deleterious effects on auditory processing and learning by allowing nonspecific sensory experiences to distort existing cortical representations.

## 4 Mechanisms of juvenile and adult plasticity

**Fig. 2:**
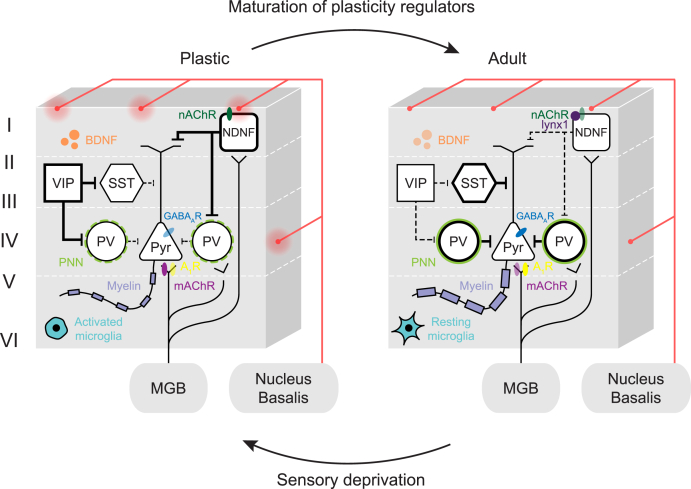
**Cellular**, **molecular, and circuit-levelmechanisms of cortical plasticity.** Regulators of plasticity are shared by the juvenile and adult auditory cortex. Cortical development is marked by changes in inhibitory circuitry and the maturation of regulators that restrict plasticity. States of heightened plasticity similar to the immature brain can be reinstated by the removal of these plasticity regulators and experiences such as learning, acoustic over-exposure, or sensory deprivation. Abbreviations: A_1_R: adenosine A1 receptor, BDNF: brain derived neurotrophic factor, GABA_A_R: GABA_A_ receptor, lynx1: ly6/neurotoxin 1 protein, mAChR: muscarinic acetylcholine receptor, MGB: medial geniculate nucleus, nAChR: nicotinic acetylcholine receptor, NDNF: neuron-derived neurotrophic factor, PNN: perineuronal net, PV: parvalbumin, Pyr: pyramidal neuron, SST: somatostatin. VIP: vasoactive intestinal peptide.

The findings above (Section 3) demonstrate the ability for altered sensory inputs to drive cortical reorganization in the adult auditory cortex. Understanding the molecular, cellular and circuit mechanisms for this heightened plasticity may provide insight into therapeutic strategies to either prevent or induce the re-wiring of aberrant cortical circuits beyond early life. Among these mechanisms, the maturation of several molecular and structural brakes serves to close CPs, notably through the developmental increase of inhibitory transmission and structural barriers to plasticity. In this section, we focus on the mechanisms that underlie experience-dependent and deprivation-induced plasticity in the developing and mature auditory cortex. We draw on parallel findings from the visual and somatosensory domains, which have also demonstrated the brain’s capacity to reopen a state of heightened plasticity following alteration of sensory inputs. Importantly, similar molecular mechanisms appear to be shared during CP and adult plasticity and are illustrated in Fig. 2.

### 4.1 Disinhibition

The onset of sensory experience triggers the maturation of inhibition (Kandler, 2004), which may act to suppress spontaneous activity generated by the cochlea prior to hearing in favor of sensory-evoked activity (Toyoizumi et al., 2013). A concurrent increase in brain-derived neurotrophic factor (BDNF) contributes to the development of adult-like auditory cortical representations (Anomal et al., 2013). BDNF may promote the maturation of GABAergic interneuron populations, including parvalbumin (PV)-expressing interneurons, heavily implicated in visual CP plasticity (Hensch, 2005; Huang et al., 1999; Itami et al., 2007). Indeed, boosting inhibition using benzodiazepines (Fagiolini et al., 2004, Fagiolini and Hensch, 2000, Hensch et al., 1998), stimulating the maturation of inhibitory circuits with BDNF (Hanover et al., 1999), or activating transcription factors enriched in interneurons (Beurdeley et al., 2012; Durand et al., 2012; Krishnan et al., 2015; Spatazza et al., 2013) can trigger CP onset in the visual cortex.

Reducing inhibitory transmission appears sufficient to revert the mature cortex to a state of juvenile plasticity permissive of changing cortical connections (Morishita and Hensch, 2008). In auditory cortex, inactivating PV cells using a chemogenetic approach permits CP-like tonotopic reorganization (Cisneros-Franco and de Villers-Sidani, 2019). Likewise, ocular dominance plasticity in visual cortex can be reactivated by decreasing GABA-mediated inhibition through fluoxetine treatment (Maya Vetencourt et al., 2008) and pharmacological inhibition of the GABA-synthesizing enzyme GAD or GABA_A_ receptors (Harauzov et al., 2010; Kuhlman et al., 2013). Enriched environments may also restore plasticity through reduced GABAergic inhibition (Sale et al., 2007).

Both juvenile and adult hearing loss are associated with acute and long-term decreases in cortical inhibition. A loss of acoustic input during development (Kotak et al., 2005; Mowery et al., 2015; Sarro et al., 2008; Takesian et al., 2010, 2012) or adulthood (Balaram et al., 2019; Browne et al., 2012; Llano et al., 2012) leads to decreased inhibition in primary auditory cortex, indicated by reduced expression of GABAergic markers, such as GAD67, GABAα1 and GABAβ2/3 receptors, reduced synaptic inhibitory currents, and reduced sensitivity of cortical networks to GABA_A_ receptor blockade. Aging has also been associated with downregulated inhibitory markers in the rodent primary auditory cortex, including reduced GAD expression, GABA_A_ receptor subunit protein levels and binding, and PV-positive and somatostatin (SST)-positive cell densities (Burianova et al., 2009; Caspary et al., 2013; Kamal et al., 2013; Ouda et al., 2008; Ouellet and de Villers-Sidani, 2014). This age-related decrease in inhibition is likely to be compounded by a natural degeneration of the auditory periphery and hearing loss associated with aging (Caspary et al., 2008, Ouda et al., 2015, Recanzone, 2018)

Reductions in PV interneuron-mediated inhibition have been implicated in cortical disinhibition following adult acoustic deprivation. Within hours after mild or severe loss of auditory nerve fibers, a drop in PV interneuron-mediated inhibition is observed in the adult mouse primary auditory cortex (Resnik and Polley, 2017). This reduced inhibition is sustained for weeks, even as hearing thresholds and cortical receptive field tuning returns to pre-exposure levels. Moreover, this early dip in PV interneuron-mediated inhibition predicts the eventual recovery of sound-evoked cortical responses weeks later. A similar rapid decrease in PV interneuron activity has been reported in the primary visual (Kuhlman et al., 2013) and somatosensory (Gainey et al., 2018) cortices following sensory deprivation. Interestingly, SST interneurons in primary auditory cortex show increased spontaneous and tone-evoked firing rates after acoustic trauma and may, therefore, serve to counteract cortical hyperexcitability (Novak et al., 2016). A better understanding of how the distinct populations of cortical inhibitory interneurons dynamically respond in the days and weeks following the onset of hearing loss will provide important insight into the mechanisms underlying cortical hyperexcitability and its behavioral consequences.

Recent work has begun to uncover the role of specific disinhibitory microcircuits gated by neuromodulatory mechanisms for cortical plasticity (Froemke et al., 2007). The most superficial cortical layer (layer 1) is sparsely populated by a diverse group of GABAergic interneurons that express the serotonin 5HT_3A_ receptor (5HT_3A_R) and are distinct from the two other commonly studied interneuron classes expressing PV or SST (Lee et al., 2010). This interneuron group is uniquely positioned to influence cortical plasticity as layer 1 receives both sound-driven inputs from the auditory thalamus and signals from several neuromodulatory systems, including the serotonergic and cholinergic systems (Lee et al., 2010; Letzkus et al., 2011; Takesian et al., 2018). Although they produce the inhibitory neurotransmitter GABA, subsets of these interneurons have a ‘disinhibitory’ function: because they target other inhibitory interneurons, their activity leads to a net withdrawal of inhibition from glutamatergic neurons (Fu et al., 2014; Letzkus et al., 2011; Pfeffer et al., 2013; Takesian et al., 2018). This disinhibitory circuit mediates tonotopic map reorganization during the CP, likely by inhibiting PV neurons (Takesian et al., 2018). Superficial 5HT_3A_R-positive interneurons expressing neuron-derived neurotrophic factor (NDNF) also play a role in plasticity that underlies adult associative learning (Abs et al., 2018). Furthermore, manipulating the activity of another group of superficial 5HT_3A_R -positive interneurons expressing vasoactive intestinal peptide (VIP) modulates plasticity in adult primary visual cortex by inhibiting SST interneurons (Fu et al., 2014). Together, these studies highlight a diverse group of disinhibitory neurons in superficial cortex as a promising target to regulate adult plasticity across sensory cortices.

### 4.2 Lifting the brakes on structural plasticity: perineuronal nets (PNNs) and myelin

Additional brakes on CP plasticity are applied by perineuronal nets (PNNs) and myelin, each of which may act as structural barriers to plasticity by prohibiting the formation of new synapses (Bavelier et al., 2010; McGee et al., 2005; Sorg et al., 2016). PNNs are extracellular matrix structures that preferentially form around PV cells, coinciding with the maturation of auditory response properties (Friauf, 2000) and potentially limiting future PV cell plasticity (Takesian and Hensch, 2013). *In vitro* degradation of PNNs reduces excitability and gain in cortex (Balmer, 2016). *In vivo* degradation of PNNs in the mature brain results in decreased inhibition (Lensjo et al., 2017) and a reopening of ocular dominance plasticity in the adult visual cortex (Lensjo et al., 2017; Pizzorusso et al., 2002). PV interneurons and PNNs may also regulate each other as part of a dynamic feedback loop: work in the adult mouse visual cortex has shown that silencing the activity of PV interneurons induces the selective regression of their own PNNs (Devienne et al., 2019).

Less clear is the role of sensory deprivation in adulthood on the dynamic regulation of PNNs. In the adult auditory cortex, a marked cell type- and layer-dependent decrease in PNNs in the mouse primary auditory cortex occurs following noise-induced hearing loss (Nguyen et al., 2017). However, similar findings have not been observed in other sensory cortices. Notably, whisker trimming during the CP disrupts formation of PNNs in the somatosensory (barrel) cortex. In contrast, whisker trimming in adulthood does not cause a loss of PNNs, suggesting that the maintenance of established PNNs does not require normal sensory input (McRae et al., 2007). Similar observations have been reported in the lateral geniculate nucleus, the thalamic relay in the visual pathway (Sur et al., 1988).

The maturation of myelin is one of the last steps of development, with central auditory myelination in both humans and rodents beginning with the onset of hearing and continuing until the age of sexual maturity (Long et al., 2018). Myelin and myelin-associated proteins, such as neurite growth inhibitors, are known to slow axon growth and limit experience-dependent plasticity in adult sensorimotor and visual cortices (Kartje et al., 1999; McGee et al., 2005; Z’Graggen et al., 1998). A growing area of research is beginning to demonstrate the role of early sensory experience and neural activity in the developmental time course of myelination (Chorghay et al., 2018). For example, musical training has been found to increase white matter connectivity in musicians who receive training before the age of 7 (Steele et al., 2013). Decreased density of myelin basic protein has been observed in young adult rats chronically exposed to white noise (Kamal et al., 2013), aged rats (de Villers-Sidani et al., 2010; Kamal et al., 2013), and aging humans (Itoyama et al., 1980). A reduction in patterned sensory inputs have, therefore, been proposed to exacerbate age-related alterations in myelin expression (Tremblay et al., 2012).

### 4.3 Synaptic remodelling: synaptogenesis, synapse unsilencing, and synaptic scaling

In the adult brain, analysis of the visual cortex points to *de novo* formation of synapses as a mechanism underlying restoration of activity in areas of deprived peripheral input. In seminal studies, focal binocular retinal lesions resulted in the deprived area of the primary visual cortex, termed the lesion projection zone, to initially become quiescent. Subsequently, however, the area regained activity and responded to loci neighboring the lesion (Gilbert et al., 1990; Kaas et al., 1990). The recovery of functional processing has been attributed to an increase in axonal boutons and axonal processes with subsequent synaptogenesis (Darian-Smith and Gilbert, 1994; Keck et al., 2008; Yamahachi et al., 2009). However, the extent of axonal sprouting appears spatially limited, such that the center of the lesion projection zone remains silent even after peripheral parts regained visually driven activity (Hubener and Bonhoeffer, 2014).

In the primary auditory cortex, the contribution of axonal rearrangement to the restoration of sound-evoked activity has not been resolved and, in fact, is contradicted by a study examining expression of various proteins in the primary auditory cortex of rats following unilateral noise exposure (Browne et al., 2012). Although various markers of inhibitory transmission were reduced in expression, a marker of axonal sprouting, GAP43, showed reduced expression in the ipsilateral auditory cortex and no change in the contralateral auditory cortex, suggesting that reorganization of the frequency map may not rely on axonal rearrangement. Alternative mechanisms, such as the unmasking of silent synapses and Hebbian strengthening of weak synapses (Syka, 2002) as well as neuronal adaptation and both short- and long-term potentiation (Froemke and Schreiner, 2015) may be involved in reorganization of cortical frequency maps following auditory deprivation. The rapid onset of reorganization suggests that unmasking is facilitated by disinhibition in the short term (Eggermont, 2017b; Scholl and Wehr, 2008), whereas Hebbian plasticity could serve to consolidate representations.

Prolonged perturbations of neuronal activity may eventually result in sensory map reorganization through homeostatic plasticity mechanisms (reviewed in Eggermont, 2017b, Gourevitch et al., 2014, Zhao et al., 2016). In cases of sensory deprivation, homeostatic plasticity serves to maintain central activity in response to decreased activity from the periphery. First described in neocortical cell cultures (Turrigiano et al., 1998), homeostatic synaptic scaling counteracts an initial loss of activity following visual deprivation in rodent (Keck et al., 2013) and human visual cortex (Castaldi et al., 2020). The same has been demonstrated in auditory cortex following conductive hearing loss (Teichert et al., 2017) and traumatic noise exposure (Asokan et al., 2018). This compensatory plasticity may result from elevated gene expression of excitatory AMPA receptors and reduced expression of inhibitory GABA_A_ receptors (Balaram et al., 2019). During chronic, non-traumatic sound exposure, homeostatic plasticity acts in the opposite manner, serving to reduce cortical activity in a frequency-specific manner in order to compensate for a persistent increase in neural activity due to narrow-band or broadband stimulation (Lau et al., 2015, Pienkowski and Eggermont, 2011).

The unsilencing of NMDA-receptor-only synapses is also a critical mechanism for CP plasticity in sensory cortices (Huang, 2019). The CP for tonotopic plasticity is associated with the unsilencing of these synapses in mouse auditory cortex. This unsilencing can occur prematurely in response to early seizures, which subsequently prevent CP reorganization (Sun et al., 2018). Severe hearing loss during early development is also associated with the elimination of AMPA-mediated long-term potentiation (LTP) (Kotak et al., 2007). LTP at auditory thalamocortical synapses can be unmasked in adulthood through attentional mechanisms or stimulation of cholinergic inputs to the cortex (Chun et al., 2013; Froemke et al., 2013), as described in more detail in the following section (section 4.4).

Microglia also contribute to synaptic remodelling during juvenile and adult cortical plasticity, especially following peripheral injury or acoustic trauma. These immune cells of the central nervous system can change from a ‘resting’ to ‘activated’ phenotype during periods of neuroinflammation (Soulet and Rivest, 2008). In both developmental and adult plasticity, microglia contribute to circuit refinement by engulfing synaptic elements such as axonal terminals and dendritic spines (Paolicelli et al., 2011; Schafer et al., 2012; Tremblay and Majewska, 2011). Increased neural activity can trigger greater microglial surveillance and modification of surrounding environments under inflammatory conditions (Wu et al., 2015), such as peripheral denervation (Ladeby et al., 2005) or traumatic noise exposure (Saljo et al., 2001). A recent study reported an increase in activated microglia in the adult auditory cortex of mice following noise-induced hearing loss (Wang et al., 2019).

### 4.4 Neuromodulatory mechanisms: linking learning and attention to map plasticity

Learning-induced changes in adult tonotopic maps are thought to require the precisely-timed release of neuromodulators recruited by arousal or attention (for reviews see Pienkowski et al., 2011; Schreiner and Polley, 2014). For example, pairing tones with mild aversive shocks causes rapid shifts in the tuning of neurons within auditory cortex toward the tone frequency (Abs et al., 2018; Bakin and Weinberger, 1990; Edeline et al., 1993). Intensive training on a sensory task can also induce dramatic changes in cortical tuning and reorganization of tonotopic maps (Bieszczad and Weinberger, 2010; David et al., 2012; Polley et al., 2006; Recanzone et al., 1993; Reed et al., 2011).

Neuromodulatory contributions to auditory cortex may also underlie the reopening of CPs in adulthood. Cholinergic modulation of plasticity has been documented in seminal experiments pairing stimulation of the nucleus basalis, the largest source of cholinergic projections to the auditory cortex, with passive tone exposure, resulting in over-representation of the exposure frequency (Froemke et al., 2007, 2013; Kilgard and Merzenich, 1998; Weinberger, 2003). Cholinergic inputs to cortical layer-1 interneurons may promote cortical plasticity by disinhibition of PV interneurons during developmental CPs (Takesian et al., 2018) and adulthood (Letzkus et al., 2011). Acetylcholine may also gate cortical plasticity by acting directly on thalamocortical synapses. Bidirectional long-term synaptic plasticity at thalamocortical synapses is gated in the mature brain by adenosine, which blocks glutamate release (Blundon and Zakharenko, 2013; Blundon et al., 2011; Chun et al., 2013). Releasing this molecular brake by deleting adenosine receptors or the adenosine synthesizing-enzyme enables map plasticity (Blundon et al., 2017). Nucleus basalis stimulation releases acetylcholine at these synapses, which, through binding to muscarinic (metabotropic) acetylcholine receptors, blocks adenosine signalling and restores CP-like plasticity (Blundon et al., 2011; Chun et al., 2013).

The noradrenergic system has also been shown to be critical for CP plasticity in the auditory cortex: pure-tone exposure does not lead to tonotopic reorganization in mice that lack norepinephrine from birth (Shepard et al., 2015), similar to seminal findings in visual cortex (Bear and Singer, 1986; Kasamatsu and Pettigrew, 1976). Indeed, pairing tones with electrical stimulation of the locus coeruleus, a brain region that releases noradrenaline, leads to shifts in frequency tuning in A1 towards the tone frequency . These findings indicate that neuromodulatory systems generally play a permissive role in CP plasticity and that the tightened control of neuromodulatory action with maturation is a main factor in restricting further experience-dependent plasticity (Morishita et al., 2010; Takesian et al., 2018). Interestingly, basal cholinergic cell numbers and cholinergic neurotransmission have long been known to decline in the aged brain (McGeer et al., 1984; Perry, 1980) and likely contribute to dysregulated auditory plasticity in later life (Caspary et al., 2008).

Together, these studies suggest that numerous interlinked mechanisms for plasticity exist at various synaptic and cellular sites within auditory cortex. Both in the developing and mature cortex, many of these mechanisms converge to influence the function of specific inhibitory circuits. Identifying circuit-level approaches to modulate plasticity will be important to prevent or reverse the maladaptive plasticity associated with adult hearing loss.

## 5 Plasticity in subcortical auditory structures

The auditory cortex receives input from the cochlea via a series of relays in the auditory brainstem. The first is the cochlear nucleus (CN), which has two main structural and functional divisions: the ventral and the dorsal CN. The dorsal CN relays directly to the inferior colliculus (IC), whereas the ventral CN first relays to the superior olivary complex (SOC), thought to be specifically involved in the binaural integration of acoustic input and sound localization. Projections from the SOC again lead primarily to the IC. From the IC, auditory signals are relayed to the medial geniculate body (MGB) in the thalamus, and, from there, to the primary auditory cortex. Subcortical auditory relays are thought to play an important role in the binaural processing of sound, as well as the integration with non-auditory information (for a more complete overview, see, for example, Malmierca et al., 2010).

During auditory CPs, neurons in these relays undergo important morphological and physiological changes that are dependent on auditory input and enable fast, reliable neurotransmission. Complete and moderate hearing loss causes changes along the length of the auditory pathway (reviewed in Butler and Lomber, 2013) that can even result in reorganization of tonotopic maps also present in the auditory brainstem (reviewed in Kandler et al., 2009). Perhaps because of their crucial role in the binaural integration of auditory input, unilateral compared to bilateral hearing loss can exert more profound effects on organization of the auditory brainstem (for example Popescu and Polley, 2010). Although the auditory brainstem was originally believed to be resistant to plasticity in adulthood to enable the stable conveyance of information to the auditory cortex, a variety of recent work in both animals and humans shows that altered acoustic input can trigger behaviorally meaningful plasticity in the adult auditory brainstem. Recent reviews have outlined various aspects of auditory brainstem plasticity (including Friauf et al., 2015; Oertel et al., 2019; Skoe and Chandrasekaran, 2014; Tzounopoulos and Kraus, 2009). Here, we highlight molecular, cellular, and circuit-level mechanisms of plasticity that are shared between the auditory cortex and auditory brainstem.

### 5.1 Inhibitory circuits

Subcortical inhibitory circuits serve to sharpen auditory responses to rapidly varying signals (reviewed in Bender and Trussell, 2011; Pollak et al., 2002). Profound changes in the balance of excitation and inhibition occur during development (reviewed in Sanes et al., 2010), and auditory deprivation during CPs disrupts maturation of inhibition along the length of the auditory pathway (reviewed in Takesian et al., 2009). These changes may reflect homeostatic mechanisms to compensate for the loss of peripheral input. A variety of likely co-occurring molecular mechanisms give rise to increased excitatory gain and decreased inhibitory gain. For example, congenitally deaf mice show enhanced intrinsic neuronal excitability in principle neurons of the medial nucleus of the trapezoid body (MNTB) due to alterations in ion channel expression (Leao et al., 2004a, 2006), enhanced excitatory (glutamatergic) synaptic transmission at endbulbs of Held, the specialized synapses between auditory neurons and bushy cells in the CN (Oleskevich et al., 2004), and diminished inhibitory (glycinergic) synaptic transmission at calyx of Held synapses between bushy cells and MNTB principle cells (Leao et al., 2004b, 2005). A similar loss of inhibitory gain following deafness in adulthood is indicated by increased expression of excitatory glutamate receptors and reduced expression of inhibitory GABA and glycine receptors in various auditory brainstem structures, including the CN (Asako et al., 2005; Dong et al., 2010a; Suneja et al., 1998, 2000), the SOC (Buras et al., 2006; Suneja et al., 1998, 2000), and the IC (Balaram et al., 2019; Dong et al., 2010a, 2010b; Holt et al., 2005). Age-related hearing loss is also associated with reduced inhibition in various structures of the auditory brainstem (reviewed in Caspary et al., 2008). These findings suggest that preventing the loss of inhibition or enhancing inhibitory circuits in the auditory pathway may mitigate auditory deficits associated with hearing loss. Indeed, recent work has shown that augmenting inhibitory neurotransmission in the thalamocortical pathway prevented perceptual defects induced by transient hearing loss during the auditory CP in gerbils (Mowery et al., 2019).

### 5.2 PNNs and myelin

PNNs and myelin have been studied in the auditory brainstem, with both appearing at the onset of hearing and progressively maturing up the neuraxis to cortex (reviewed in Long et al., 2018; Sonntag et al., 2015). PNNs, in general, surround fast and precisely spiking neurons and thus are, not surprisingly, found in subsets of neurons in almost all brainstem auditory relays. They are especially prominent around octopus cells, principal cells that fire with high temporal precision in the ventral CN, and around principle cells in the MNTB (Sonntag et al., 2015). Genetic attenuation of PNNs in MNTB principle neurons reduces their excitability and alters their firing properties (Balmer, 2016; Blosa et al., 2015). Neonatally deafened rats show reduced expression of PNN markers in neurons of the medial superior olive (Myers et al., 2012). These findings motivate further work to examine the functional consequences of altered PNNs in response to auditory deprivation and modulation during developmental CPs and in adulthood.

Myelination is essential for rapid conduction and precise timing of action potentials. A recent study found that, in the mouse, both axonal diameter and myelin thickness of neurons in the MNTB double two weeks after the onset of hearing, with a concurrent (and expected) increase in transmitted firing rates and conduction speed (Sinclair et al., 2017). In the same study, peripheral auditory deprivation via ear plugging at hearing onset led to comparatively thinner and less myelinated fibers, and ear plugging in adulthood also reduced myelin thickness, most notably in the most myelin-rich fibers. In rats, acoustic overexposure in adulthood causes, among other pathologies, disorganization of myelin sheaths around axon fibers of auditory neurons (Coyat et al., 2019). The molecular mechanisms regulating myelination either normally or in response to altered acoustic input are unknown. Recent work examining knockout mice indicates that β-secretase 1 (BACE1), a protease shown to regulate myelination and myelin sheath thickness in central and peripheral nerves, may regulate auditory-nerve myelination, at least during development (Dierich et al., 2019).

### 5.3 Synaptic plasticity

During auditory CPs, neurons of the auditory brainstem undergo dramatic morphological rearrangements that are dependent on auditory input (reviewed in O’Neil et al., 2011; Yu and Goodrich, 2014). This structural maturation is necessary for fast and reliable synaptic transmission, and is especially evident in the endbulbs of Held found in the CN. Ascending processes of the auditory nerve form progressively more arborized synaptic endings, called endbulbs of Held, which eventually envelop the cell bodies of the bushy cell neurons in the anterior ventral CN (Ryugo and Fekete, 1982). Auditory deprivation during development causes profound morphological changes in the maturation of these contacts (Baker et al., 2010), with similar, albeit less dramatic, changes also occurring with progressive, age-related hearing loss (Connelly et al., 2017) and hidden hearing loss caused by cochlear synaptopathy (Muniak et al., 2018). Specifically, endbulbs show overall reduced branching with hypertrophy of the remaining postsynaptic densities in both cats (Baker et al., 2010) and mice (Lee et al., 2003). This hypertrophy appears related to the degree of hearing loss: cats with congenital hearing loss (thresholds above 50 dB SPL) exhibit synapses with sizes between those of totally deaf cats and normal-hearing cats (Ryugo et al., 1997). In addition, in congenitally deaf cats, hypertrophy of the endbulb synapses is more evident in spherical compared to globular bushy cells, whereas synapses of bouton endings onto multipolar cells remain unaltered (Redd et al., 2002). The hypertrophy of synapses on spherical bushy cells, where ionotropic glutamate receptors are located, may serve to compensate reduced input from the auditory nerve. Consistent with this hypothesis, hypertrophy of the postsynaptic densities is significant in spherical bushy cells, which show high levels of spontaneous activity, modest in globular bushy cells, which show more medium levels of spontaneous activity, and absent in multipolar cells, which show medium-to-low levels of spontaneous activity (Redd et al., 2000, 2002; Ryugo et al., 1997, 1998). Importantly, early restoration of hearing via cochlear implants in congenitally deaf cats rescues normal synaptic structure in both the CN (O’Neil et al., 2011; Ryugo et al., 2005) and in the medial SOC (Tirko and Ryugo, 2012). Thus, restoration of input early in the ascending auditory pathway is crucial to preventing the cascade of pathological plasticity observed along the length of the auditory pathway in response to both early and late deafening.

The molecular factors regulating morphological changes of auditory synapses in response to auditory deprivation during developmental CPs and in adulthood are still unclear. In response to auditory deprivation, the CN shows increased expression of pro-apoptotic genes during auditory CPs in contrast to increased expression of pro-survival genes following CPs (Harris et al., 2005). Researchers examining gene expression changes in the ventral CN of adult rats shortly after noise exposure highlighted decreased expression of *Bdnf*, *Homer*, and *Grin1*. These genes encode proteins involved in neurogenesis and plasticity and may, therefore, play a role in synaptic remodelling (Manohar et al., 2019). Previous work has also implicated growth-associated protein 43 (GAP43), a brain-wide regulator of synapse structural and functional plasticity (Holahan, 2017) and BDNF (Singer et al., 2014) in synaptic remodelling of neurons in the CN. Re-expression of GAP43 can be induced by unilateral cochlear ablation of neurons in the ipsilateral CN (Fredrich and Illing, 2010; Illing et al., 1997; Kraus et al., 2011) and ipsilateral lateral superior olive (Illing et al., 1997). GAP43 expression is maintained into adulthood in the IC and lateral and medial superior olivary nuclei (Illing et al., 1999), suggesting retained potential for plasticity into adulthood. Finally, increased and prolonged expression of activated microglia is associated with acoustic overexposure (Baizer et al., 2015), consistent with the role of microglia in repair after neuronal injury.

### 5.4 Neuromodulatory mechanisms

Although the role of acetylcholine as a neuromodulator regulating plasticity has been better studied in the auditory cortex (see Section 4.4), the cochlea and many of the intervening auditory relays to the cortex also receive cholinergic innervation that regulates plasticity. Both nicotinic and muscarinic cholinergic receptors are extensively expressed throughout the auditory brainstem (Morley and Happe, 2000), reflecting the potential for widespread influence of cholinergic signalling along the auditory pathway. In the auditory brainstem, cholinergic innervation arises from neurons in the SOC and pontomesencepahlic tegmentum, which form not only ascending but also collateral and descending projections within the auditory brainstem (reviewed in Schofield et al., 2011). These two structures also receive innervation from the auditory cortex (Schofield et al., 2011). Particularly well-studied are the primarily cholinergic olivocochlear projections from the SOC (specifically the medial and lateral superior olives) to the cochlea (reviewed in Fuchs and Lauer, 2019). Medial olivocochlear neurons receive input from the CN and provide inhibitory output to cochlear outer hair cells, creating a feedback loop that is thought to be important for dynamic improvement of hearing in background noise. The pontomesencepahlic tegmentum targets the MGB in the thalamus, the IC, and the CN (Schofield et al., 2011). Although diverse effects of acetylcholine on responses of neurons in the IC have been reported (Schofield et al., 2011), cholinergic modulation appears to exert more profound effects on sound-evoked (rather than spontaneous) activity and appears to increase excitability by causing a release of inhibition (Ayala et al., 2016). In the thalamus, a recent study showed that activation of nicotinic acetylcholine receptors on MGB neurons enhances both ascending inhibitory (tectothalamic) projections and descending excitatory (corticothalamic) inputs, potentially integrating bottom-up and top-down mechanisms to improve signal detection (Sottile et al., 2017). Finally, thalamocortical plasticity is malleable not only during auditory CPs (Chun et al., 2013), but can also be unmasked by activation of muscarinic cholinergic inputs to the thalamus (Blundon et al., 2011). In addition to acetylcholine, glutamate, GABA, glycine, and dopamine also regulate plasticity in the auditory brainstem as part of descending pathways (reviewed in Schofield and Beebe, 2019). Improved understanding of the role of these various neuromodulatory mechanisms will allow manipulation of anatomically and functionally specific circuits to regulate plasticity in the auditory brainstem to counteract perceptual defects induced by hearing loss.

## 6 Hearing loss, (maladaptive) plasticity in the auditory pathway, and the emergence of tinnitus

Noise- and age-related hearing loss are the biggest risk factors for tinnitus (Elgoyhen and Langguth, 2010; Shore et al., 2016), the perception of phantom sounds, which affects between 12 and 30% of adults (McCormack et al., 2016; Møller, 2011). In animals, hearing loss has been associated with changes in cortical response properties (see Section 3), suggesting that cortical plasticity induced by hearing loss may maladaptively trigger tinnitus. A causal relationship between hearing loss and tinnitus through maladaptive plasticity is supported, not only by the high comorbidity of hearing loss and tinnitus, but also by the observation that the perceived frequency profile of the phantom percept often coincides with the frequency profile of the hearing loss (Norena et al., 2002; Schecklmann et al., 2012). However, hearing loss is not always associated with tinnitus (Møller, 2011), and individuals with normal audiograms (but perhaps hidden hearing loss) can also report tinnitus (e.g., Schaette and McAlpine, 2011). Much research is currently focused on disentangling the neural changes associated with tinnitus from those associated strictly with hearing loss. Additional, albeit more limited, work is focused on disentangling the confound of aging on both tinnitus and hearing loss (reviewed in Ibrahim and Llano, 2019).

Early research implicated reorganization of the tonotopic map in the auditory cortex as a potential neural correlate of tinnitus in humans (e.g., Mühlnickel et al., 1998) and animals (e.g., Eggermont and Roberts, 2004; Irvine, 2000; Kaltenbach, 2011). For example, pure tone exposure causing mild to moderate high-frequency hearing loss—often associated with tinnitus in humans (Tan et al., 2013)—led to profound reorganization of the cortical tonotopic map in cats (Eggermont and Komiya, 2000). One study suggested a direct causal relationship between tonotopic map reorganization and tinnitus, showing that tone exposure paired with vagal-nerve stimulation both reversed tonotopic map reorganization and eliminated behavioral indicators of tinnitus in previously noise-exposed rats (Engineer et al., 2011). However, very recent research indicates that cortical reorganization is not necessary for tinnitus. By examining mouse strains with different susceptibilities to developing tinnitus, Miyakawa and colleagues showed that cortical map distortions were associated with hearing loss but not tinnitus (Miyakawa et al., 2019). Importantly, research with animal models is complicated by the varied methods used to induce tinnitus and the lack of consensus on reliable measures to detect its presence (reviewed in von der Behrens, 2014).

In humans, brain imaging (functional magnetic resonance imaging, fMRI) studies have found no evidence of cortical tonotopic reorganization when examining subjects with tinnitus but normal or near-normal hearing thresholds (Berlot et al., 2020; Langers et al., 2012). A recent study that used subjects with or without tinnitus but otherwise carefully matched for moderate to profound high-frequency hearing loss found that changes in cortical responses were associated with hearing loss and were, in fact, more pronounced in hearing loss without than with tinnitus (Koops et al., 2020). The direction of the changes, however, was opposite to that predicted from the animal literature, with increased responses to frequencies within—rather than outside of—the hearing loss range (i.e., increased responses to higher frequencies). Koops et al. (2020) presented their stimuli at equal loudness levels across subjects, such that stimulus intensities were greater in individuals with greater hearing loss. Thus, it is possible that the observed differences between hearing-impaired subjects with and without tinnitus reflect differences in the peripheral pattern of the hearing loss (Tan et al., 2013) rather than changes arising centrally as a consequence of hearing loss. Consistent with this idea, earlier studies, which used constant stimulus levels, found either no changes in cortical responses associated with hearing loss (Lanting et al., 2008; Wolak et al., 2017) or increased responses to lower frequencies (Ghazaleh et al., 2017), opposite to the findings of Koops et al. (2020). Importantly, research in humans is complicated by the heterogeneity of tinnitus causes and presentation (reviewed in Cederroth et al., 2019).

Lack of cortical reorganization in humans with tinnitus is consistent with brain imaging studies in the visual and somatosensory domains. In the visual domain, macular degeneration is a condition which, like high-frequency hearing loss, deprives a limited part of the cortical sheet of its input. fMRI studies have shown that, even after years of visual input deprivation due to macular degeneration, the relevant cortical regions remain non-responsive (e.g., Baseler et al., 2011; Smirnakis et al., 2005; Sunness et al., 2004), suggesting that cortical organization is stable despite input deprivation. In fact, somatosensory studies have suggested that cortical stability and phantom perception are causally related. These studies exploited the fact that patients with limb amputations often experience vivid impressions that they can move the missing limb. They showed that such phantom perceptions of missing limb movements can elicit orderly activations within the deprived cortical regions (Kikkert et al., 2016) and that the strength of the activation is associated with the severity of phantom limb pain (Kikkert et al., 2018; Makin et al., 2013). Thus, findings across sensory domains suggest that phantom percepts may arise, not from cortical map reorganization, but, conversely, from map stability.

As another strategy to disentangle the effects of hearing loss and tinnitus, new lines of research have begun to examine the contribution of presumably non-traumatic sound experience—potentially causing hidden forms of hearing loss—to changes in cortical responses (reviewed in Eggermont, 2017a) as well as tinnitus. Cats exposed to either synthetic tone pips or white noise (Pienkowski and Eggermont, 2009), or to “real-world” noise (Pienkowski et al., 2013) at non-traumatic levels demonstrated altered neural activity in the auditory cortex, suggesting that passive sound exposure may lead to a tinnitus percept in the absence of overt hearing loss (reviewed in Pienkowski and Eggermont, 2012). Independent lines of research indicate that presumably non-traumatic noise exposure can lead to substantial and permanent loss of synapses between the inner hair cells and auditory neurons—called cochlear synaptopathy—that is not detectable by audiograms (reviewed in Liberman and Kujawa, 2017), raising the possibility that tinnitus may be triggered by audiometrically hidden forms of hearing loss such as cochlear synaptopathy. Findings testing this prediction have so far been inconsistent, with some work suggesting that synaptopathy is associated with tinnitus (Forster et al., 2018; Rüttiger et al., 2013) and other work finding no link (Hickox and Liberman, 2014; Pienkowski, 2018). Both biological and experimental differences (e.g., strain and species differences and methods of tinnitus detection) may account for the variability in findings.

Research in animals has implicated a few molecular candidates and pathways that appear specifically involved in the generation of tinnitus following hearing loss. Downregulation of GAD65, an enzyme required in the synthesis of the inhibitory neurotransmitter GABA, is necessary for the generation of tinnitus following noise-induced hearing loss (Miyakawa et al., 2019). Decreased mobilization of Arc/Arg3.1, an activity-dependent cytoskeletal protein required for homeostatic synaptic plasticity in mouse primary visual cortex (Gao et al., 2010; Sedley et al., 2015), in the auditory cortex is associated with the development of tinnitus, but not hearing loss alone, following noise exposure (Rüttiger et al., 2013). Genetic knockout and pharmacological blockade of TNFα, a proinflammatory cytokine also involved in regulating homeostatic plasticity (Steinmetz and Turrigiano, 2010; Stellwagen and Malenka, 2006), prevented noise-induced generation of tinnitus; conversely, TNFα administration caused tinnitus behavior in normal-hearing animals (Wang et al., 2019). Together these findings indicate that there are molecular factors that regulate whether (or not) plasticity triggered by hearing loss results in tinnitus.

Although cortical map organization may be stable despite input deprivation, there is consensus that tinnitus is associated with altered neural activity and cortical plasticity in some form. Metabolic measures, such as fMRI, may be insufficient to detect these types of plasticity, as they cannot distinguish between excitatory versus inhibitory activity (Heeger and Ress, 2002; Logothetis, 2008) and may, therefore, fail to detect changes in inhibition following auditory deprivation that have been observed in animals (e.g., Norena and Eggermont, 2003; Rajan, 1998). Consistent with this idea, results from human studies using magnetic resonance spectroscopy (MRS) have suggested that both hearing loss (Gao et al., 2015) and tinnitus (Sedley et al., 2015) can be associated with reductions in the concentration of the main inhibitory neurotransmitter, GABA, within the auditory cortex. Similar changes have also been observed in the visual cortex as a result of visual deprivation (Lunghi et al., 2015).

In animals, the observation of altered neural activity in association with noise-induced tinnitus is not restricted to the auditory cortex, and, in fact, has been more extensively studied in subcortical structures of the auditory pathway. Here too, research efforts are focused on identifying the cellular and molecular changes that distinguish hearing loss with tinnitus from hearing loss alone (reviewed in Shore and Wu, 2019). Cortical structures also provide descending corticofugal input, which is thought to play a role in cortical plasticity and learning (reviewed in Malmierca and Ryugo, 2010; Suga, 2008; Suga, 2012) and may, therefore, also regulate the generation of tinnitus. In humans, tinnitus has been found to be associated with both increased (Boyen et al., 2014; Lanting et al., 2008) and decreased (Hofmeier et al., 2018) activity in subcortical structures, as well as altered connectivity between cortical and subcortical structures (Hofmeier et al., 2018; Husain and Schmidt, 2014).

Finally, in both animals and humans, tinnitus has been linked to hyperactivity in non-auditory brain areas (reviewed in Middleton and Tzounopoulos, 2012), as well as altered connectivity between non-auditory and auditory brain areas (reviewed in Husain and Schmidt, 2014). Therefore, the maintenance of the tinnitus percept may involve a distributed network of brain areas, including those involved in limbic, memory and attentional function, which have previously been thought to control the emotional/cognitive response to tinnitus (reviewed in Elgoyhen et al., 2015; Shahsavarani et al., 2019). Additional work is necessary to clarify the contributions of these subcortical and non-auditory structures to plasticity of the auditory cortex associated with tinnitus and to distinguish the structures that generate tinnitus from those that maintain it.

## 7 Conclusion

In the adult brain, a complex interplay of cellular, molecular, and circuit-level mechanisms governs cortical plasticity in response to altered acoustic experience and hearing loss, with likely distinct mechanisms giving rise to hearing loss with tinnitus. Importantly, work in animals has shown that adult plasticity requires extrinsic alterations and possible recruitment of neuromodulatory circuits with constraints imposed by intrinsic (genetic) programs. In humans, there appear to be not only individual differences in the susceptibility to tinnitus, which may result from intrinsic genetic differences (Cederroth et al., 2017), but also individual differences in the response to tinnitus treatment (McFerran et al., 2019). These treatments, which currently rely on various approaches and combinations of approaches, including hearing aids, sound therapy, brain stimulation, cognitive and behavioral therapy, medication, and acupuncture (Bauer et al., 2017; Brennan-Jones et al., 2019; Lin et al., 2019), may activate pathways regulating plasticity. Therefore, a more precise understanding of the mechanistic interplay of factors regulating plasticity will enable the development of more effective, reliable, and personalized treatments for tinnitus.
